# Supplementary material for: CNAViz: An interactive webtool for user-guided segmentation of tumor DNA sequencing data
Source: PLoS Comput Biol. 2022 Oct 13;18(10):e1010614. doi: 10.1371/journal.pcbi.1010614 (PMC9595559; doi:10.1371/journal.pcbi.1010614)
Supplement: Attachment — Submitted filename: response.pdf [file pcbi.1010614.s003.pdf]

Point-by-point response

Reviewer #1

I reviewed this paper for RECOMB so I am reviewing it in the spirit of a revision even if it is technically a new submission to PLOS CB. Overall, I retain some of my original concerns about the appropriate criteria for evaluating a visualization tool in this space, but I believe the authors do show the software to be useful to the community. They have been very responsive to the critiques of the conference version and I believe have addressed the criticisms pretty much as well as would be possible without making it a substantially different contribution. They have added a lot of new features to the software and new demonstrations and clarifications, as well as improved documentation and instructional videos. The nature of the work still raises some concerns about whether it is computational enough for a computational biology forum as well as whether it is adequately assessed by the standards of a visualization paper. The authors might reconsider in revision whether any more can be done to address those points. But I believe the work is meritorious despite these concerns and the authors make a reasonable argument not to do more on those issues. I do not have any new concerns to raise at this point and am satisfied that the contribution is sound.

We thank the reviewer for their detailed and thoughtful comments which have led to improvements in the paper and tool.

Reviewer #2

General:

CNAViz is a webtool that allows the user to visualize and manually correct the segmented copy number sequencing data for whole genome DNA bulk sequencing. The user can also perform de novo manual segmentation. The graphical interface makes the tool accessible even for unexperienced users thanks to the user manual that not only explains how to use CNAViz, but also how to prepare the data for the analysis. The authors clearly justify why such manual inspection and correction of copy-number segmentation is needed in the process of analyzing CNAs in cancer.

Major:

1. The authors acknowledge the existence of two very different types of DNA sequencing data - bulk and single cell. This is indeed a very important subject and I believe that the authors should state explicitly, preferably in the abstract, whether CNAViz in the current form is applicable to both, or only one of those two types of DNAseq data.

As more nuance is required in the discussion of using single cell data with CNAViz, we have added a lengthy discussion on the matter in the Availability and Future Directions section (see below).

Additionally, in the paragraph with the overview of existing CN calling algorithms, the methods for bulk sequencing should be separated from scDNAseq methods.

We have separated out the sentences discussing the methods concerning single cell and bulk sequencing data.

The single cell data suitable for CN calling is significantly different from bulk- for example the coverage for whole genome scDNAseq is incomparably lower (usually less than 0.05X) and RDR or BAF info is not easily obtainable. It is my understanding that CNAViz is right now a tool for bulk DNAseq data and this should be repeated in this paragraph together with a detailed overview of relevant existing methods for bulk. scDNAseq should of course be acknowledged, but in a separate sentence.

We separate out the discussion of the bulk and single cell methods in the Introduction:

> "Examples of these algorithms for DNA sequencing data include ASCAT, BIC-seq}, Control-FREEC, TITAN for bulk tumor samples. Additionally, the methods HMMcopy and Ginkgo have been developed for single cell DNA sequencing data."

If the authors want to dwell into existing methods for CN calling in scDNAseq data, they should also explain that it presents different challenges and the possibility of addressing them by CNAViz. This is touched upon in the discussion, which I believe is the correct place, but should be expanded on since the scalability is not the only and the biggest challenge for inferring CNs in scDNAseq.

In the Availability and Future Directions section, we expound on the difficulties of inferring copy numbers on single cell DNA data. We further discuss the current limitations of CNAViz on the task of visualizing single-cell DNA sequencing data.

> "Compared to bulk DNA sequencing data, the lower coverage in single-cell data results in fewer bins that span larger genomic regions (e.g. the single-cell data illustrated in Figure D in S1 Text has 5MB bins as compared to 50 KB bins in bulk whole genome sequencing data). However, the main challenge is that the number of samples in single cell data, which can be as large as 1,000 cells, far exceeds CNAViz's capacity for effective visualization and comparisons across samples."

> "Thus, although CNAViz can be used to visualize single-cell DNA sequencing data, it will likely require some changes to improve the analysis across samples. Moreover, we

> envision the interface to aid the user to detect doublets as well as determine cell-specific scaling factors used in downstream copy-number calling. We leave this to future work. Finally, we propose an opt-in way for users to contribute segmentation solutions akin to crowd-sourcing efforts like FoldIt, enabling future developments of automated segmentation algorithms that incorporate successful strategies employed by expert users."

If I am mistaken and the current version of CNAViz is applicable to scDNAseq, at least one example of such analysis should be given.

We discuss the role which CNAViz plays with single cell DNA sequencing data in the Availability and Future Directions section, and provide one example of a visualization in the Supplement.

> "Third, while this manuscript focused on applications of CNAViz to bulk DNA sequencing data, CNAViz is also applicable to single cell DNA sequencing data. We refer the reader to Figure D in S1 Text for an example."

Minor:

1. The switching back to "Zoom" Mode does not work after I have used the "Select" mode. Sometimes I need to clear the browsing data and cookies and then refresh the page to be able to use the "Zoom" again.

Unfortunately, we were unable to reproduce this problem. However, we changed the "Zoom" and "Pan" modes so that the mouse scroll zoom works in both modes.

2. The name of "Erase" mode is not accurate as it may suggest that is removes clustering. I would simply change to "Deselect".

We have made this change. All references to this button should now refer to it as the Deselect button, and we've changed the icon to match the select button.

3. The abstract would benefit from removing of repetition of "important" in the following sentence: "Importantly, inaccurate segmentation will lead to inaccurate identification of important CNAs.

Done.

4. In the abstract, is: “To improve copy-number segmentation and their control”, should be: „To improve copy-number segmentation and its control”.

We removed “and their control”.

5. Lines 8 and 9, is: Not only is the identification of CNAs key to understanding cancer evolution”, should be „Not only is the identification of CNAs a key to understanding cancer evolution”.

Done.

6. Lines 24 and 25, is: „Thus, analyzing variations of RDR and BAF values across bins allow the identification”, should be: „Thus, analyzing variations of RDR and BAF values across bins allows the identification”.

Done.

7. Line 258, is: “users can choose a cluster I”, should be: „users can choose a cluster i”.

We have taken “i” out, since “cluster i” is not referenced again.

8. Lines 262 to 264 – I would explicitly name the buttons on the sidebar and inform the user that „Undo cluster” also works for reversing the quite powerful “Clear clustering” button. Also “Clear clustering” should work locally i.e. clear clustering for only the selected area.

We changed the tool tip for the “Undo Cluster” button to indicate that it includes the “Clear Clustering” action. We also added a pop-up to ask for user confirmation when “Clear Clustering” is clicked, explaining that this will remove all of the current clustering.

We believe that clearing the clustering locally would overcomplicate the task for the user, as the clustering is shared across bins. Instead, we encourage users to select the local clustering and manually clear the clustering by assigning those bins to -1.

9. Line 294, is: “and restricted ourselves”, should be: “and restrict ourselves”.

Done.

10. Line 296, is: “genomic region that the driver gene spans is highlighted.”, should be: „ genomic region of the driver gene is highlighted.”

Done.

11. Line 297, should be Figure 2j.

Done.

12. Some problems with how the axis text is double printed in the purity/ploidy mode.

We have made changes to the user interface so that all axes, both local and global, are responsive. When there are overlaps between axis ticks, only some will be shown, so that no text is double printed.

13. Line 310, is “Any pair”, should be: “All pairs”.

Done.

14. Line 313, is “which, should be “that”.

Done.

15. Line 393, is “from”, should be “From”.

Done.

We thank the reviewer for their detailed and thoughtful comments which have led to improvements in the paper and tool.
